# A Novel Probable Pathogenic PSEN2 Mutation p.Phe369Ser Associated With Early-Onset Alzheimer's Disease in a Chinese Han Family: A Case Report

**DOI:** 10.3389/fnagi.2021.710075

**Published:** 2021-07-21

**Authors:** Ke Wan, Zhen-Juan Ma, Xia Zhou, Yi-Mei Zhang, Xian-Feng Yu, Meng-Zhe You, Chao-Juan Huang, Wei Zhang, Zhong-Wu Sun

**Affiliations:** Department of Neurology, The First Affiliated Hospital of Anhui Medical University, Hefei, China

**Keywords:** early-onset Alzheimer's disease, PSEN2, magnetic resonance imaging, transmembrane domain 7, novel p.Phe369Ser mutation

## Abstract

The pathogenesis of Alzheimer's disease is complex, and early-onset Alzheimer's disease (EOAD) is mostly influenced by genetic factors. Presenilin-1, presenilin-2 (PSEN2), and amyloid precursor protein are currently known as the three main causative genes for autosomal dominant EOAD, with the PSEN2 mutation being the rarest. In this study, we reported a 56-year-old Chinese Han proband who presented with prominent progressive amnesia, aphasia, executive function impairment, and depression 5 years ago. The 3-year follow-up showed that the patient experienced progressive brain atrophy displayed on magnetic resonance imaging (MRI) and dramatic cognitive decline assessed by neuropsychological evaluation. This patient was clinically diagnosed as EOAD based on established criteria. A heterozygous variant (NM_000447.2: c.1106T>C) of PSEN2 was identified for the first time in this patient and her two daughters. This mutation causing a novel missense mutation (p.Phe369Ser) in transmembrane domain 7 encoded by exon 11 had not been reported previously in 1000Genomes, ExAC, or ClinVar databases. This mutation was predicted by four *in silico* prediction programs, which all strongly suggested that it was damaging. Our results suggest that this novel PSEN2 Phe369Ser mutation may alter PSEN2 protein function and associate with EOAD.

## Introduction

Alzheimer's disease (AD) is a neurodegenerative disease characterized by cognitive decline, which can be categorized as early-onset AD (EOAD) and late-onset AD (LOAD). EOAD, which accounts for 5–10% of all AD cases, is diagnosed with an age at onset (AAO) before 65 years old (Tellechea et al., 2018). Although the etiology of AD remains unknown, mutations in three autosomal dominant causative genes, presenilin-1 (PSEN1), presenilin-2 (PSEN2), and amyloid precursor protein (APP), have been frequently reported to be associated with EOAD. To date, 17 pathogenic mutations related to AD have been gradually identified in the PSEN2 gene (https://www.alzforum.org/mutations), which is less frequently reported than PSEN1 (274 AD-related pathogenic mutations) and APP (27 AD-related pathogenic mutations). PSEN2 plays an essential role in the pathogenesis of AD by mediating the cleavage of APP via γ-secretase in the generation of amyloid β-protein (Aβ) (Wilkins and Swerdlow, 2017) and increasing the high intracellular Aβ 42/40 ratio (Sannerud et al., 2016).

In this study, we first identified a novel PSEN2 p.Phe369Ser mutation from a Chinese Han family, and its pathogenicity was predicted by four software programs, which suggested that it might be associated with EOAD. We followed this patient for 3 years by comparing the progression of her cerebral atrophy using structural magnetic resonance imaging (MRI) combined with cognitive and neuropsychological assessments.

## Materials and Methods

### Subject

**Figure 1:**
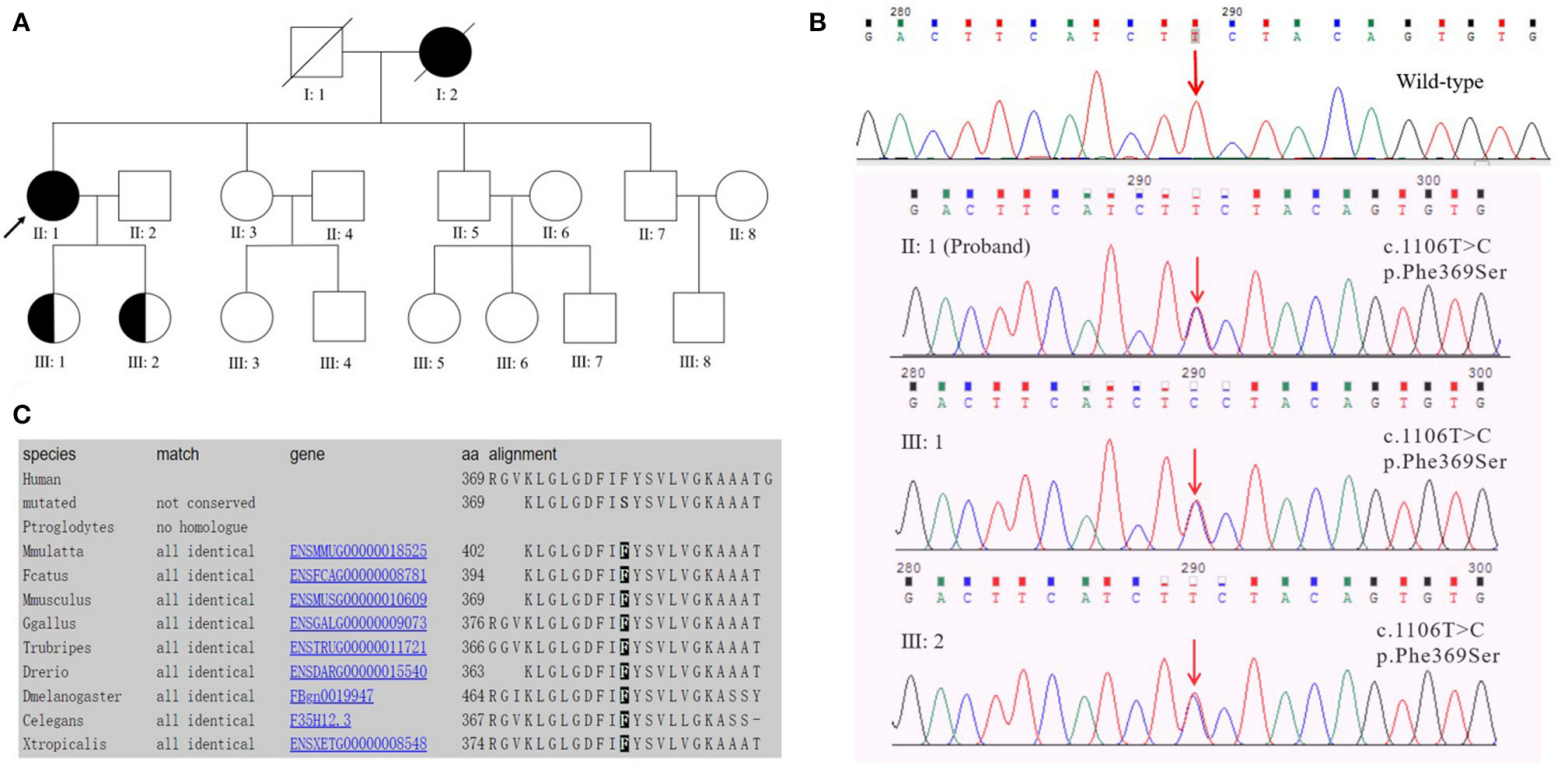
**(A)** Pedigree of early-onset Alzheimer's disease with the PSEN2 Phe369Ser mutation. The proband is marked with an arrow; circles and squares represent females and males, respectively; filled symbols, affected family members; half-filled symbols, mutation carriers (currently with no clinical symptoms of EOAD). Blood samples were available from the proband (II: 1), her sister (II: 3), brothers (II: 5, II: 7), and her daughters (III: 1, III: 2). **(B)** DNA sequencing chromatography indicated a heterozygous PSEN2 c.1106T>C mutation (nomenclature according to NCBI. Reference Sequence: NM_000447.2) in the proband and her two daughters. **(C)** Evolutionary conservation analysis of phenylalanine (Phe or F) at position 369 among different species.

The proband (Figure 1A II: 1) was initially recruited in the outpatient clinic of the Department of Neurology in the First Affiliated Hospital of Anhui Medical University in August 2018. The diagnosis of probable AD was based on the 2011 National Institute on Aging and Alzheimer's Association criteria (McKhann et al., 2011). This study was approved by the Ethics Committee of the First Affiliated Hospital of Anhui Medical University, and signed informed consent was obtained from the patient and her legal guardians.

### Brain Structural MRI Processing

Three-year brain 3D-T1 weighted images (3D-T1WI) of this proband were analyzed via the Computational Anatomy Toolbox for SPM12 (http://www.neuro.uni-jena.de/cat/) in MATLAB R2014b (The MathWorks Inc, Massachusetts, USA). This toolbox consists of bias-field and noise removal, skull stripping, segmentation into gray matter (GM), white matter, and cerebrospinal fluid. MRI images were normalized and registered to a 1.5 mm isotropic adult Montreal Neurological Institute space template using Diffeomorphic Anatomical Registration Through Exponentiated Lie Algebra. Then, the GM images were segmented and smoothed using a Gaussian kernel with a full width at half maximum of 8 mm, with the GM volume measured.

### Cognitive and Neuropsychological Assessments

From 2018 to 2020, the same neuropsychological assessments were performed yearly by an identical neuropsychological technician within 1 day after the brain MRI scan on this patient. The tests included the Mini-Mental State Examination (MMSE), Montreal Cognitive Assessment (MoCA), Cambridge Cognitive Examination-Chinese version (CAMCOG-C), Geriatric Depression Scale (GDS), Activities of Daily Living Scale (ADL), Clinical Dementia Rating (CDR), Hamilton Anxiety Scale (HAMA), and Hamilton Depression Scale (HAMD).

### Blood Test

Blood samples were collected from the proband (II: 1) and the proband's five first-degree relatives (except for her parents who had died), including her sister (II: 3), brothers (II: 5, II: 7) and daughters (III: 1, III: 2) (Figure 1A). Laboratory examinations were performed on the subjects' blood samples by standard laboratory techniques, including routine blood examination, serum biochemicals, blood lipid tests, thyroid hormone levels, C-reactive protein, human immunodeficiency virus antibody and Treponema pallidum tests, homocysteine, folic acid, and vitamin B12.

### Target Region Capture and Genetic Analysis

The QIAamp Blood Mini Kit (Qiagen, Hilden, Germany) was employed to extract genomic DNA from peripheral blood leukocytes. A dementia-related gene panel (see Supplementary Material) was designed to capture all exons, splice sites, and immediately adjacent intron sequences. The analysis gene panel contained 53 candidate genes associated with cognitive impairment. Genomic DNA integrity was assessed by agarose gel electrophoresis, and the DNA was quantitated and analyzed by NanoDrop2000 and Qubit3.0. Genome fragmentation was carried out using an M220 Focused-Ultrasonicator (Covaris, Woburn, Massachusetts, USA), and the fragmented DNA was terminally repaired by adding an A base at the 3' end to connect the joints. The DNA target region was hybrid-captured using the XGen Exome Research Panel v1.0 kit (IDT, USA). The captured and amplified DNA samples were sequenced using the Illumina NovaSeq 6000 System (Illumina, San Diego, CA, USA). After the sequencing data were evaluated by Sequence Control Software (Illumina, USA), data reading and bioinformatics analysis were performed. The pathogenic gene mutation sites of patients were screened out through the above capture sequence and validated using the Sanger sequencing method. The apolipoprotein E (APOE) genotype was identified by the real-time quantitative PCR-high resolution melting curve (qPCR-HRM) method using a LightCycler 96 instrument (Roche Diagnosis, Germany).

### *In silico* Predictions

The identified mutation was determined via 1000Genomes (http://browser.1000 genomes.org/), Exome Aggregation Consortium (ExAC, https://gnomad.broadinstitute.org/) and ClinVar (https://www.ncbi.nlm.nih.gov/clinvar/) databases. The pathogenicity of this variant was predicted by PolyPhen-2, SIFT, PROVEAN, and Mutation Taster prediction tools. SWISS-MODEL (https://swissmodel.expasy.org/) was used to acquire PDB files of both wild-type and mutant PSEN2 gene sequences and predict the 3D structure conformations. The PyMOL Molecular Graphics System version 2.4.1 (http://pymol.org) was used to superimpose the 3D structure of PDB files between wild-type and mutant PSEN2.

## Case Report

### The Proband and Family Medical History Description

**Figure 2:**
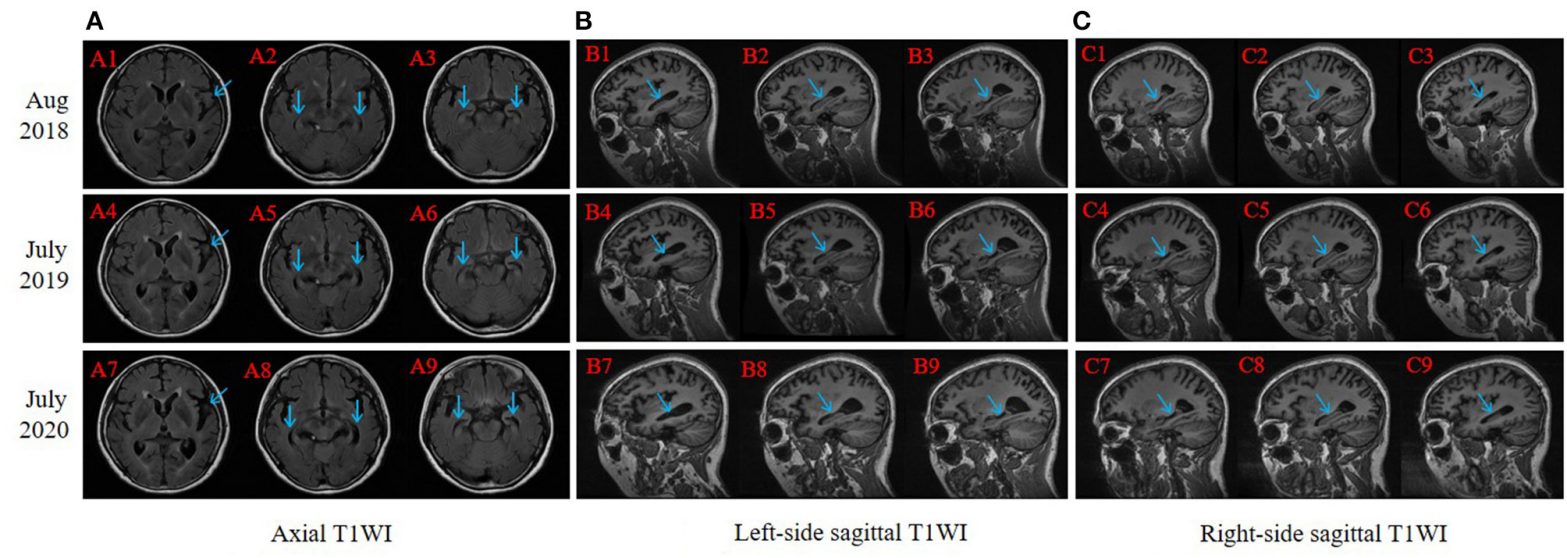
Brain magnetic resonance imaging (MRI) slice-corresponding comparison of the proband for 3 years. Arrows point at progressive atrophy of temporal lobe and hippocampus. A1–A3, B1–B3, and C1–C3 were scanned in August 2018 (2 years after dementia symptom onset in this proband); A4–A6, B4–B6, and C4–C6 were scanned in July 2019; and A7–A9, B7–B9, and C7–C9 were scanned in July 2020. **(A)** Axial T1WI: each column revealed progressive atrophy of the left-dominant bilateral temporal lobe and hippocampus. **(B)** Left-side sagittal T1WI: focused on the left part of the hippocampus. **(C)** Right-side sagittal T1WI: focused on the right part of the hippocampus.

The right-handed proband was a 56-year-old female with at least 8 years of education. At the age of 51, she had initially presented with progressive forgetfulness, mainly with a notable decline in recent memory. She usually forgot what she had just said and repeated the same sentences, did not remember where she put things, and could not find things she wanted. She had a poor date and place orientation. In addition, she had difficulty concentrating, reacting, and comprehending information, along with a distinct depressive and anxious condition. Later, her personality gradually changed, with unstable emotions, reduced speech, and the inability to find correct and complete words to express herself. She was afraid of going out alone and became very dependent on her guardians. She could not handle her money or purchase items. Additionally, she could not well perform the movements she used to be familiar with; for instance, she was a barber, yet she did not know how to use scissors anymore. Though she managed to accomplish some household chores, she made many mistakes during these procedures. During the follow-up years, her clinical manifestations progressively worsened. In the latest evaluation, she could not recognize others except for her guardians or find her way home and failed to understand some simple questions when we conducted the last evaluation. Her past medical history revealed only hypertension for 5 years. At present, her blood pressure is under control with proper medical treatment. She had no disturbance of gait, spasticity, ataxia, or any Parkinsonian features. Nervous system examination presented no motor or sensory problems and a negative pathologic reflex. The results of routine laboratory tests showed that all the indicators were within the reference ranges. Her cognitive and neuropsychological assessments at 2 years after symptom onset indicated the existence of anxiety, depression, impairment in activities of daily living, with the follow-up assessments showing dramatic decline (see Table 1). Her electroencephalogram (EEG) showed that the main activity was low and medium potential 4-7c/s θ waves, accompanied by a very small number of 8-9c/s α waves, which may further support the diagnosis of AD (Schmidt et al., 2013). Her brain MRI revealed progressive atrophy of the left-dominant bilateral temporal lobe and hippocampus (Figure 2). Her GM volume decreased consistently from a baseline of 440 cm^3^, through 392 cm^3^ at the second visit, to 365 cm^3^ at the third visit (see Table 1). The patient was then treated with donepezil (10 mg QD), memantine (10 mg BID) and paroxetine (20 mg QD).

**Table 1:** Detailed changes in the cognitive and neuropsychological assessments and brain total GM volume of the proband during the 3-year follow-up.

**Items**	**1st visit (Aug, 2018)**	**2nd visit (July, 2019)**	**3rd visit (July, 2020)**
Age (y)	53	54	55
MMSE	10/30	9/30	3/30
MoCA	8/30	7/30	4/30
CAMCOG-C	44/107	38/107	8/107
Orientation	3/10	2/10	0/10
Language	18/30	15/30	4/30
Comprehension	8/9	5/9	1/9
Expression	10/21	10/21	3/21
Memory	4/27	4/27	0/27
Remote memory	0/6	0/6	0/6
Recent memory	0/4	0/4	0/4
Learning memory	4/17	4/17	0/17
Attention	1/7	1/7	0/7
Praxis	8/12	7/12	2/12
Calculation	1/2	1/2	0/2
Abstraction	3/8	2/8	0/8
Perception	6/11	6/11	2/11
GDS	16/30	15/30	5/30
ADL	33/80	44/80	61/80
CDR	2/3	2/3	3/3
HAMA	N	11/56	16/56
HAMD	N	4/68	4/68
GM volume (cm^3^)	440	392	365

*MMSE, Mini-Mental State Examination; MoCA, Montreal Cognitive Assessment; CAMCOG-C, Cambridge Cognitive Examination-Chinese version; GDS, Geriatric Depression Scale; ADL, Activities of Daily Living Scale; CDR, Clinical Dementia Rating; HAMA, Hamilton Anxiety Scale; HAMD, Hamilton Anxiety Depression; GM, gray matter. N, not applicable*.

The proband had a positive family history (Figure 1A). The proband's mother had similar clinical symptoms of short-term memory loss at the age of 60. As the illness deteriorated, the proband's mother could not recognize anyone and exhibited abnormal psychiatric behavior. Finally, the proband's mother completely lost her basic living ability and soon died before reaching the age of 70. The proband's sister (Figure 1A II: 3, 53 years old) and two brothers (Figure 1A II: 5, 50 years old; II: 7, 47 years old) exhibited normal cognitive status. The proband's two daughters (Figure 1AIII: 1, 29 years old; III: 2, 26 years old) were PSEN2 Phe369Ser mutation carriers. Both of the probands' two daughters received 30/30 on MMSE at the last visit and showed no similar clinical manifestations to the proband.

### Genetic Analysis and *in silico* Predictions

**Figure 3:**
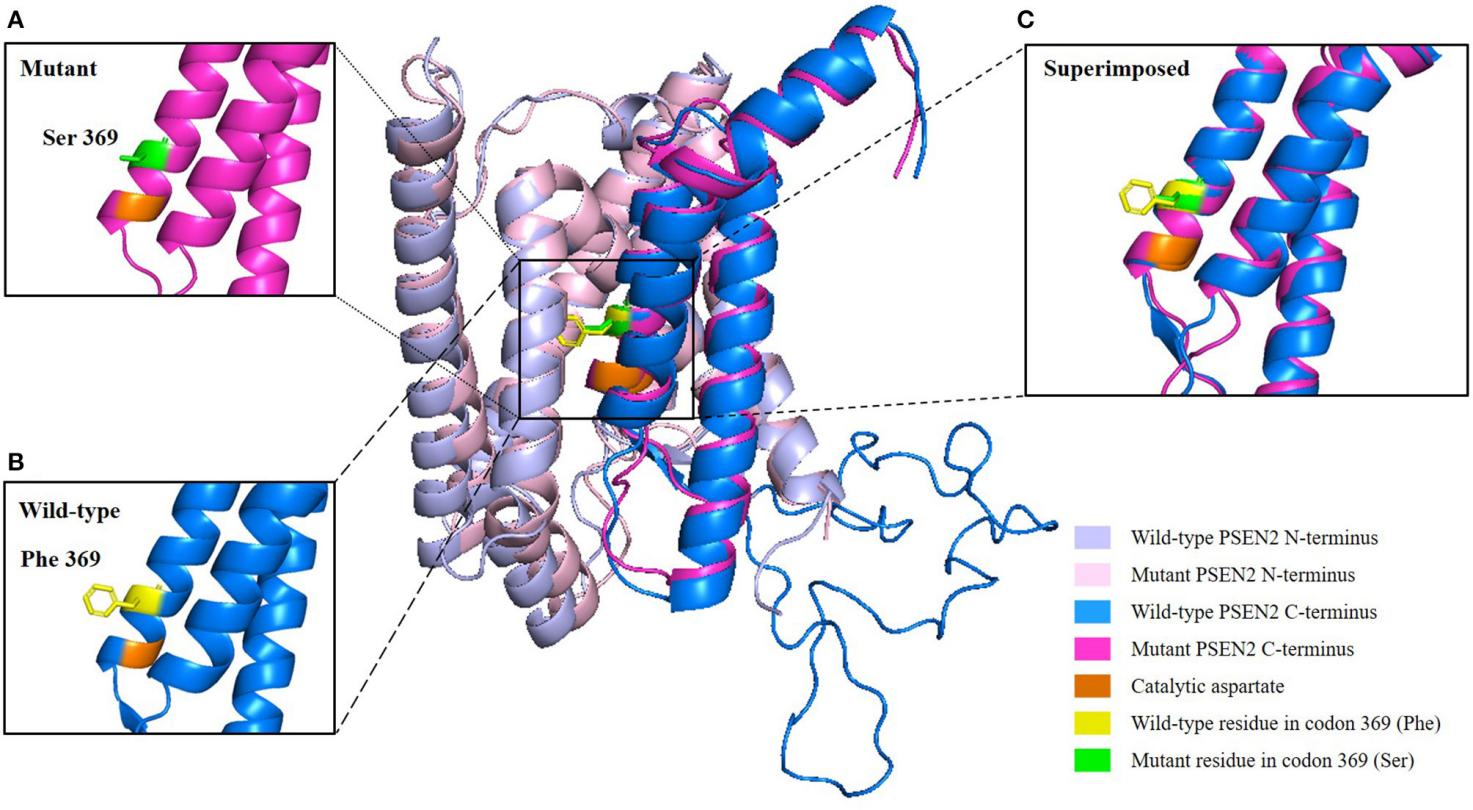
3D structure prediction of the PSEN2 p.Phe369Ser mutation. Wild-type PSEN2 N-terminus: light blue; mutant PSEN2 N-terminus: light pink; wild-type PSEN2 C-terminus: marine blue; mutant PSEN2 C-terminus: magenta. Catalytic aspartate on transmembrane domain 7 is colored orange. Wild-type and mutant residues in position 369 of PSEN2 are highlighted in yellow and green, respectively. **(A)** Zoomed-in view of the mutant PSEN2 C-terminus. **(B)** Zoomed-in view of the wild-type PSEN2 C-terminus. **(C)** Zoomed-in view of the superimposed wild-type and mutant PSEN2 C-terminus, which displayed substitution of phenylalanine by serine at codon 369 (Phe369Ser).

Target region capture sequencing uncovered a novel heterozygous missense variant (NM_000447.2: c.1106T>C) in the proband located at exon 11 of PSEN2, which caused a substitution from phenylalanine (Phe or F) to serine (Ser) at codon 369 (Phe369Ser) (Figure 1B). No variants were found in other dementia-related genes among the designed gene panel. The APOE allele of this proband and her first-degree relatives (two daughters and three siblings) was of the ε3/ε3 genotype. Both of the probands' two daughters had the same PSEN2 Phe369Ser mutation, while the other relatives were normal. This mutation had not been reported in the 1000Genomes, ExAC, or ClinVar databases. The evolutionarily conservative prediction revealed that missense mutation p.Phe368Ser led to a highly conserved amino acid change (Figure 1C). 3D protein structures of wild-type and mutant PSEN2 superimposition displayed the substitution from phenylalanine to serine at position 369 (Figure 3). Four *in silico* prediction tools consistently and strongly supported the pathogenicity of the PSEN2 p.Phe369Ser: PolyPhen-2 predicted probably damaging (both HumDiv and HumVar scores: 1.000); SIFT predicted damaging (score: 0.001); PROVEAN predicted deleterious (score: −7.23); Mutation Taster predicted disease causing. These results indicated that this novel PSEN2 mutation p.Phe369Ser was the most likely pathogenic cause of EOAD.

The genome sequence was submitted in NCBI Sequence Read Archive (SRA) database, under the name of “PSEN2 Mutation p.Phe369Ser” (TaxID 9606), Biosample accession number: SAMN19277214, BioProject accession number: PRJNA731321, Submission ID: SUB9688990, and Locus tag prefix: KJK35. The project information is accessible on publication from http://www.ncbi.nlm.nih.gov/bioproject/731321.

## Discussion

In this study, we first identified a novel PSEN2 p.Phe369Ser mutation in an EOAD Chinese Han family. *In silico* prediction tools and 3D-protein structure modeling strongly predicted the pathogenicity of this variant that might affect the function of PSEN2.

This proband in our study exhibited typical clinical symptoms of EOAD, including AAO before 65 years of age, progressive memory loss, and deteriorated cognitive impairments. Three-year MRI showed continuous atrophy, and EEG indicated increased θ waves and decreased α waves without any etiology to cause dementia. Nevertheless, her two daughters, who both carried the same genetic mutations, were in normal cognitive status. According to previous studies (Lleó et al., 2002; Jayadev et al., 2010), AAO ranged from 39 to 80 years for patients with PSEN2 mutations, and the Volga German pedigrees (N141I mutation) had a mean AAO of 53.7 (±7.8) years, with a mean disease duration of ~10 years. The mean AAO of pedigrees with PSEN2 in China was 61.47 (±8.71) years (Jia et al., 2020), which was older than that of PSEN1 and APP kindreds. This Chinese epidemiological study also unveiled that APOE ε4 seemed to be a promoter of the clustering of familial AD without PSEN2 mutations. In our study, the proband and her two daughters (APOE ε3/ε3) had PSEN2 mutations at the same time, but only the proband was clinically diagnosed with AD at present. These findings indicate that there are some complicated interactions of both genetic and environmental factors in the clinical phenotype of EOAD. We have kept contact with this pedigree and have conducted regular cognitive follow-ups with the proband and carriers. Based on the evidence above, we presume that this variant may be pathogenic and related to EOAD.

The PSEN2 protein, as the catalytic component of γ-secretase complexes, is assembled with additional proteins, including anterior pharynx defective 1, nicastrin and presenilin enhancer 2 (Oikawa and Walter, 2019). γ-Secretase is a complicated tetrameric membrane-embedded protease (Yang et al., 2019), which plays a significant role in the pathogenesis of AD by modulating the cleavage of APP and the generation of Aβ. The Aβ peptide, the main component of amyloid plaques known as one of the neuropathological hallmarks of AD, is derived from abnormal cleavage of APP by β- and γ-secretases through the amyloidogenic pathway (Zheng and Koo, 2006).

During complex formation, the PSEN2 protein undergoes autocatalytic endoproteolysis within cytosolic hydrophilic loop 2 to generate N-terminal and C-terminal fragments, which is essential for the activity of γ-secretase (De Strooper and Annaert, 2010). The p.Phe369Ser variant is located at the conserved residue of TM-VII in the C-terminal region of the PSEN2 protein. To date, 13 different mutations, including Phe369Ser, have been reported in the C-terminus of PSEN2 (see Supplementary Material), which accounts for 20.3% (13/64) of the total PSEN2 mutations. Furthermore, the majority of these 13 mutations are damaging according to the prediction by *in silico* programs. The C-terminus protrudes into the lumen or extracellular space (Escamilla-Ayala et al., 2020) and may affect the pathological function of PSEN2 by regulating the signal for entering the endoproteolytic processing pathway (Shirotani et al., 2000). The C-terminus also contains the conserved PAL motif and the hydrophobic C-terminal tip, both of which are crucial to catalytic activity and the formation of the γ-secretase complexes (Sato et al., 2008).

PSEN2 has nine transmembrane domains (TMDs), with transmembrane domain 6 (TM-VI) and transmembrane domain 7 (TM-VII), each containing one of two catalytic aspartyl residues (Wolfe et al., 1999). Catalytic aspartates are critical for γ-secretase activity. Position Phe369 is close to one of the catalytic aspartate residues located on Asp366 of TM-VII (Figure 3). We hypothesized that substitution Phe369Ser might alter the posttranslational modification and spatial structure or interact with adjacent amino acid residues and thus influence the function of the PSEN2 protein (Xia M. et al., 2015). However, this hypothesis needs to be verified by further studies.

Previously, many studies had discussed the pathogenic mechanism of familial AD (FAD) caused by presenilin mutations. Emerging evidence supported that the presenilin hypothesis might be a better explanation for the pathogenesis of FAD compared with the alternative amyloid hypothesis (overproduction and aggregation of Aβ peptides). The presenilin hypothesis proposed that presenilin mutations could bring about loss of functions of PSEN protein, suppress γ-secretase activity, and lead to neurodegeneration and cognitive dysfunction (Shen and Kelleher III, 2007; Heilig et al., 2010; Xia D. et al., 2015). The biochemical properties of the PSEN catalytic subunit in the γ-secretase complexes influence both endopeptidase and carboxypeptidase-like activities (Acx et al., 2014). Previous studies suggest PSEN2 complexes exhibit similar endopeptidase cleavage specificities of APP but less functional carboxypeptidase-like activities compared to the PSEN1 counterparts (Bentahir et al., 2006; Acx et al., 2014). Several *in vitro* studies showed that FAD-causing PSEN1 mutations could inhibit γ-secretase activity, decreasing overall Aβ production, whereas increasing the ratio of Aβ42/40 (Sun et al., 2017; Arber et al., 2020). FAD mutations in PSEN2 also generate a high intracellular Aβ 42/40 ratio (Bentahir et al., 2006; Quintero-Monzon et al., 2011; Sannerud et al., 2016).

Human PSEN1 and PSEN2 proteins have 66% similarity of sequence homology with TMDs being the most conserved (Escamilla-Ayala et al., 2020). The PSEN2 Phe369Ser mutation is also located at the conserved TM-VII domain in the homologous PSEN1 Phe388. There are five pathogenic mutations in the near position of PSEN1 Phe388 (Phe386Ser, Phe386Ile, Phe386Leu, Tyr389Ser, Tyr389His), but there is no mutation reported previously at position 369 (Phe369Ser) or an adjacent position in the PSEN2 protein. Since PSEN2 mutations are relatively rare, studies mainly focus on PSEN1 and neglect PSEN2. To figure out whether the functions and characteristics of PSEN1 can also be applied to PSEN2, a deep comparison of PSEN2 vs. PSEN1 complexes is warranted by future functional and structural studies (Acx et al., 2014).

The strengths of this study are as follows: we performed a 3-year longitudinal follow-up of a pedigree with novel PSEN2 mutations, and multiple cognitive assessments, MRI evaluations, and genetic analyses were conducted. However, this study has several limitations. First, it lacks evidence of biomarker changes in cerebrospinal fluid, 18F-fluorodeoxyglucose positron emission tomography, and biopsy validation because the patient's guardians refused these examinations. Second, we could not obtain blood samples from this patient's parents since they had died before we first saw the proband; therefore, it is difficult to conclude whether this variant belongs to a *de novo* mutation. Third, we were unable to accomplish functional studies to further identify the possible pathogenesis of this novel mutation and its connection with EOAD. Additionally, we were not able to recruit enough matched healthy controls with a 3-year follow-up to acquire their brain 3D-T1WI results to further compare the progression of brain atrophy between the patient and normal people.

## Conclusions

In conclusion, we discovered a novel mutation in exon 11 at position 369 (Phe369Ser) of the PSEN2 gene associated with EOAD in a Chinese Han family. The 3-year follow-up of the proband showed aggravation of cognitive deterioration and progressive atrophy of the left-dominant bilateral temporal lobe and hippocampus. *In silico* prediction tools strongly indicated that this variant was probably damaging. Our results expand the gene polymorphism of rare pathogenic PSEN2 mutations; however, further functional studies are needed to identify the pathogenesis of this mutation in AD.

## Data Availability Statement

The original contributions presented in the study are included in the article/Supplementary Material, further inquiries can be directed to the corresponding author/s.

## Ethics Statement

The studies involving human participants were reviewed and approved by The Ethics Committee of the First Affiliated Hospital of Anhui Medical University. The patients/participants provided their written informed consent to participate in this study.

## Author Contributions

KW and Z-WS: study concept and design and drafting of the manuscript. XZ and Z-JM: analysis of data. Y-MZ, X-FY, and M-ZY: acquisition of MRI data. C-JH and WZ: acquisition and interpretation of assessment data. All authors read and approved the final manuscript.

## Conflict of Interest

The authors declare that the research was conducted in the absence of any commercial or financial relationships that could be construed as a potential conflict of interest.

## Abbreviations

3D-T1WI: 3D-T1 weighted images
ADL: Activities of Daily Living Scale
AAO: age at onset
AD: Alzheimer's disease
APP: amyloid precursor protein
Aβ: amyloid β-protein
APOE: apolipoprotein E
CAMCOG-C: Cambridge Cognitive Examination-Chinese version
CDR: Clinical Dementia Rating
EOAD: early-onset Alzheimer's disease
EEG: electroencephalogram
FAD: familial AD
GDS: Geriatric Depression Scale
GM: gray matter
HAMA: Hamilton Anxiety Scale
HAMD: Hamilton Depression Scale
LOAD: late-onset Alzheimer's disease
MRI: magnetic resonance imaging
MMSE: Mini-Mental State Examination
MoCA: Montreal Cognitive Assessment
Phe or F: phenylalanine
PSEN1: presenilin-1
PSEN2: presenilin-2
Ser: serine
TM-VI: transmembrane domain 6
TM-VII: transmembrane domain 7.
