# Supplementary material for: A Novel Probable Pathogenic PSEN2 Mutation p.Phe369Ser Associated With Early-Onset Alzheimer's Disease in a Chinese Han Family: A Case Report
Source: Front Aging Neurosci. 2021 Jul 21;13:710075. doi: 10.3389/fnagi.2021.710075 (PMC8334358; doi:10.3389/fnagi.2021.710075)
Supplement: Supplementary file 1 [file Data_Sheet_1.PDF]

Supplementary Material

1 Supplementary Data

Dementia-related gene panel (including 53 candidate genes) associated with currently known diseases that cause cognitive impairment is as follow:

A2M, ACE, ACHE, ADAM10, APBB2, APOE, APP, ATN1, ATP13A2, BCHE, C9orf72, CHMP2B, CHRM1, CLU, CR1, DNMT1, GBA, GRN, GSTO1, HFE, HNRNPA1, HNRNPA2B1, ITM2B, KLK1, LOC643387, MAPT, MEOX2, MPO, NOS3, NOTCH3, NPC1, PAXIP1, PICALM, PLAU, PLD3, PRNP, PSEN1, PSEN2, RETREG1, RPS27A, SLC6A4, SNCA, SNCB, SORL1, SQSTM1, TARDBP, TBK1, TNFSF14, TREM2, TRPM7, TYROBP, UBQLN2, VCP.

2 Supplementary Tables

SUPPLEMENTARY 2 | Clinical characteristics of reported mutations located in the C-terminus of PSEN2 (exon)

Mutation (NM_000447.2)	AAO (years)	Sex	Clinical diagnosis	Clinical symptoms	Atypical features	Disease duration (years)	Country/ Ethnic	APOE ε4	Family history	Pathogenicity	PolyPhen-2		SIFT (score)	PROVEAN (score)	MT	MRI	Reference
											HumDiv (score)	HumVar (score)					
p.Thr301Met (c.902C>T)	60s	M	AD	Progressive memory loss	N	>10	Netherland	N	yes	Unclear	Pos D (0.593)	B (0.067)	T (0.245)	Neu (-0.81)	D	N	(Croes et al., 2004)
p.Lys306fs (c.917delA)	55	M	EOAD	Progressive memory loss	Language impairment, aphasia, visuospatial disorientation	>6	Morocco	N	yes	Unclear	N	N	N	D (-4.29)	D	Cortical atrophy	(El Kadmiri et al., 2014)
p.Pro334Arg (c.1001C>G)	80	F	AD	Memory and cognitive disturbances	Word-finding difficulty.	5	Spain	+	yes	Not	B (0.316)	B (0.392)	T (0.293)	Neu (-0.22)	P	Cortical atrophy in temporal and parietal areas	(Lleó et al., 2002)
p.Pro334Ala (c.1000C>G)	<65	N	probable AD	N	N	<10	Caribbean Hispanic	N	yes	Unclear	B (0.040)	B (0.156)	T (0.295)	Neu (-0.75)	P	N	(Lee et al., 2014)
p.Pro348Leu (c.1043C>T)	65	F	EOAD	Progressive memory loss	Pronounced deficits of apraxia and affective indifference	8	German	N	no	Unclear	B (0.001)	B (0.008)	T (0.669)	Neu (-2.42)	D	N	(Blauwendraat et al., 2016)
p.Phe369Ser (c.1106T>C)	51	F	probable AD	N	N	>5	China	-	yes	Pathogenic	Pro D (1.000)	Pro D (1.000)	D (0.001)	D (-7.23)	D	Progressive atrophy of the left-dominant bilateral temporal lobe and hippocampus	This study
p.Ala377Val (c.1130C>T)	<65	N	probable AD	N	N	<10	Caribbean Hispanic	N	yes	Unclear	Pro D (1.000)	Pro D (1.000)	D (0.044)	Neu (-2.32)	D	N	(Lee et al., 2014)
p.Ala379Asp (c.1136C>A)	55	N	probable AD	N	N	N	China	-	no	Pathogenic	Pro D (0.996)	Pro D (0.983)	D (0.025)	D (-2.95)	D	N	(Wang et al., 2019)
p.Val393Met (c.1177G>A)	50	M	probable AD	Progressive memory disturbances, profound impairment of episodic memory	Severe language impairment early in the course of the disease	7	Denmark	+	yes	Unclear	Pro D (1.000)	Pro D (1.000)	D (0.015)	D (-2.59)	D	N	(Lindquist et al., 2008)
p.Thr421Met (c.1262C>T)	55	F	EOAD	Progressive memory loss	N	N	Japan	+	no	Pathogenic	Pro D (1.000)	Pro D (0.988)	D (0.015)	D (-4.48)	D	N	(Yagi et al., 2014)
p.Thr430Met (c.1289C>T)	45	M	probable AD	Progressive cognitive and behavioral disturbances	Myoclonus, grasping, and generalized epileptic seizures	11	Spain	-	yes	Unclear	Pro D (1.000)	Pro D (1.000)	D (0.000)	D (-5.32)	D	Discrete global atrophy	(Ezquerra et al., 2003)
p.Pro436Leu (c.1307C>T)	52	M	probable AD	Progressive memory and cognitive disturbances, personality changes	N	>4	China	+	yes	Unclear	Pro D (1.000)	Pro D (0.999)	D (0.001)	D (-8.98)	D	Atrophy of the bilateral temporal lobe and hippocampus	(Han et al., 2020)
p.Asp439Ala (c.1316A>C)	52	M	probable AD	Progressive memory and cognitive decline, behavioral changes	N	>6	Spain	-	yes	Unclear	Pro D (0.961)	Pos D (0.794)	D (0.006)	D (-6.00)	D	Moderate cortical atrophy in the frontal and parietal regions	(Lleó et al., 2001)

Key: AAO, age at onset; M, male; F, female; N, not applicable; AD, Alzheimer's disease; EOAD, early-onset AD; (-) means no APOE e4 genotype; (+) indicates the APOE e4 genotype; fs, frameshift mutation; del, deletion; MRI, magnetic resonance imaging; Pos, possibly; Pro, probably; D, damaging; B, benign; T, tolerated; Neu, neutral; P, polymorphism; MT, Mutation Taster

REFERENCES:

Blauwendraat, C., Wilke, C., Jansen, I.E., Schulte, C., Simón-Sánchez, J., Metzger, F.G., Bender, B., Gasser, T., Maetzler, W., Rizzu, P., Heutink, P., Synofzik, M., (2016). Pilot whole-exome sequencing of a German early-onset Alzheimer's disease cohort reveals a substantial frequency of PSEN2 variants. *Neurobiol. Aging* 37, 208.e211-208.e217. doi: 10.1016/j.neurobiolaging.2015.09.016

Croes, E.A., Theuns, J., Houwing-Duistermaat, J.J., Dermaut, B., Sleegers, K., Roks, G., Van den Broeck, M., van Harten, B., van Swieten, J.C., Cruts, M., Van Broeckhoven, C., van Duijn, C.M., (2004). Octapeptide repeat insertions in the prion protein gene and early onset dementia. *J. Neurol. Neurosurg. Psychiatry* 75(8), 1166-1170. doi: 10.1136/jnnp.2003.020198

El Kadmiri, N., Zaid, N., Zaid, Y., Tadevosyan, A., Hachem, A., Dubé, M.P., Hamzi, K., El Moutawakil, B., Slassi, I., Nadifi, S., (2014). Novel presenilin mutations within Moroccan patients with Early-Onset Alzheimer's Disease. *Neuroscience* 269, 215-222. doi: 10.1016/j.neuroscience.2014.03.052

Ezquerra, M., Lleó, A., Castellví, M., Queralt, R., Santacruz, P., Pastor, P., Molinuevo, J.L., Blesa, R., Oliva, R., (2003). A novel mutation in the PSEN2 gene (T430M) associated with variable expression in a family with early-onset Alzheimer disease. *Arch. Neurol.* 60(8), 1149-1151. doi: 10.1001/archneur.60.8.1149

Han, L.H., Xue, Y.Y., Zheng, Y.C., Li, X.Y., Lin, R.R., Wu, Z.Y., Tao, Q.Q., (2020). Genetic Analysis of Chinese Patients with Early-Onset Dementia Using Next-Generation Sequencing. *Clin. Interv. Aging* 15, 1831-1839. doi: 10.2147/CIA.S271222. eCollection 2020

Lee, J.H., Kahn, A., Cheng, R., Reitz, C., Vardarajan, B., Lantigua, R., Medrano, M., Jiménez-Velázquez, I.Z., Williamson, J., Nagy, P., Mayeux, R., (2014). Disease-related mutations among Caribbean Hispanics with familial dementia. *Mol. Genet. Genomic Med.* 2(5), 430-437. doi: 10.1002/mgg3.85

Lindquist, S.G., Hasholt, L., Bahl, J.M., Heegaard, N.H., Andersen, B.B., Nørremølle, A., Stokholm, J., Schwartz, M., Batbayli, M., Laursen, H., Pardossi-Piquard, R., Chen, F., St George-Hyslop, P., Waldemar, G., Nielsen, J.E., (2008). A novel presenilin 2 mutation (V393M) in early-onset dementia with profound language impairment. *Eur. J. Neurol.* 15(10), 1135-1139. doi: 10.1111/j.1468-1331.2008.02256.x

Lleó, A., Blesa, R., Gendre, J., Castellví, M., Pastor, P., Queralt, R., Oliva, R., (2001). A novel presenilin 2 gene mutation (D439A) in a patient with early-onset Alzheimer's disease. *Neurology* 57(10), 1926-1928. doi: 10.1212/wnl.57.10.1926

Lleó, A., Castellví, M., Blesa, R., Oliva, R., (2002). Uncommon polymorphism in the presenilin genes in human familial Alzheimer's disease: not to be mistaken with a pathogenic mutation. *Neurosci. Lett.* 318(3), 166-168. doi: 10.1016/s0304-3940(01)02499-5

Wang, G., Zhang, D.F., Jiang, H.Y., Fan, Y., Ma, L., Shen, Z., Bi, R., Xu, M., Tan, L., Shan, B., Yao, Y.G., Feng, T., (2019). Mutation and association analyses of dementia-causal genes in Han Chinese patients with early-onset and familial Alzheimer's disease. *J. Psychiatr. Res.* 113, 141-147. doi: 10.1016/j.jpsychires.2019.03.026

Yagi, R., Miyamoto, R., Morino, H., Izumi, Y., Kuramochi, M., Kurashige, T., Maruyama, H., Mizuno, N., Kurihara, H., Kawakami, H., (2014). Detecting gene mutations in Japanese Alzheimer's patients by semiconductor sequencing. *Neurobiol. Aging* 35(7), 1780.e1781-1785. doi: 10.1016/j.neurobiolaging.2014.01.023
